# The validity and reliability of the Arabic version of the EQ-5D: a study from Jordan

**DOI:** 10.4103/0256-4947.55313

**Published:** 2009

**Authors:** Salah Aburuz, Naela Bulatova, Mohammed Twalbeh, Moatasem Gazawi

**Affiliations:** a Department of Clinical Pharmacy, Faculty of Pharmacy, University of Jordan, Amman, Jordan; b Department of ENT, Faculty of Medicine and Jordan University Hospital, University of Jordan, Amman, Jordan

## Abstract

**BACKGROUND AND OBJECTIVES:**

EQ-5D is a generic measure that permits comparisons in quality of life across disease states, and which may provide useful data for health policy and resource allocation decision-making. There are no published reports on the acceptability and psychometric properties of the EQ-5D in the Arabic language. We therefore investigated the validity and reliability of the Arabic translation of the EQ-5D in Jordan.

**METHODS:**

The study was conducted on a convenience sample consisting of consecutive adult Arabic-speaking outpatients or visitors attending a university teaching hospital. Subjects were interviewed twice using a standardized questionnaire containing the EQ-5D, Short Form 36 Health Survey (SF-36). To assess the validity of the Arabic version of the EQ-5D, ten hypotheses relating responses to EQ-5D dimensions or the visual analogue scale (EQ-VAS) to SF-36 scores or other variables were examined and test-retest reliability was assessed.

**RESULTS:**

The study included 186 subjects who had a mean age of 45.3 years and included 87 (47%) females. The major problem reported in more than 102 (55%) of the subjects was anxiety/depression. All of the ten a-priori hypothesis relating EQ-5D responses to external variables were fulfilled. Cohen's κ for test-retest reliability (n=52) ranged from 0.48 to 1.0.

**CONCLUSION:**

The Arabic translation of EQ-5D appears to be valid and reliable in measuring quality of life in Jordanian people.

Measurement of health-related quality of life (HRQL) has become an imperative in clinical trials and disease management programs for a variety of diseases. HRQL can be considered a major outcome in clinical trials in the absence of objective measures. In Jordan, where Arabic is the first language, conducting clinical trials is difficult due to the lack of validated Arabic translations or versions of quality of life instruments. The EQ-5D is a standardised generic instrument for use as a measure of health and quality of life outcomes. Applicable to a wide range of health conditions and treatments, it provides a simple descriptive profile and a single index value for health status. The EQ-5D is now available in most major languages with cultural adaptations.^1^–^3^ There are no published reports on the acceptability and psychometric properties of the EQ-5D iin the Arabic language, which is the first language for more than 300 million people. Using a convenience sample of lay people we therefore investigated the validity and reliability of the Arabic version of the EQ-5D in Jordan as a prelude to a future population-based valuation of health states. Jordan is a small country in the Middle East with a population of over 5 million people. This study is part of a larger project aiming at developing, translating and adapting important instruments and questionnaires into Arabic.

## METHODS

The study group was a convenience sample that consisted of consecutive adult Arabic-speaking outpatients or visitors attending the University of Jordan hospital in the period between June to August 2007. The inclusion criteria were that the subject should be an adult Jordanian and should have no obvious cognitive deficit. The hospital is located at the center of Amman, the capital of Jordan, and is considered one of the oldest and largest hospitals in Jordan serving more than 0.5 million people every year. The study site was ideal for testing the instrument as it serves a diverse group of patients from all over the country.

Translation and cross-cultural adaptation was done according to the EuroQol group's guidelines.^4^ Two independent translators performed forward translation, followed by backward translation by another two translators. When the consensus version was determined, cognitive debriefing was done by ten laypersons. They underwent a structured interview to assess understandability and ease of completion of the Arabic EQ-5D.

The translation process was smooth and straightforward. The translators only disagreed on the translation of “discomfort” during the forward translation process as it can be translated into several closely related words. A discussion session was conducted until the translators agreed on the most appropriate translation. During the interview with the ten laypersons, they reported no concerns with the phrasing of the Arabic EQ-5D. In general, the instrument was very easy to complete and clear to all readers.

The study was approved by the University of Jordan Academic and Research Committee. All participants were interviewed by trained research assistants using a questionnaire containing the Arabic version of the EQ-5D and the short form health survey (RAND SF-36-Arabic version).^5^ Demographic data were also collected using a standard questionnaire. A random sample was then chosen, given a copy of the EQ-5D and re-interviewed over the phone using the EQ-5D after 2 to 4 weeks to assess test-retest reliability.

The EQ-5D6 consists of a self classifier with five single item health dimensions, each with three response levels, and a visual analogue scale (EQ-VAS). Both the health state descriptors and the visual analogue scale of the perceived health state of the Arabic version of the EQ-5D were used in the current study. The SF-36 is a validated,^5^,^7^,^8^ 36-item instrument measuring perceived health in eight dimensions with higher scores (range 0 to 100) reflecting better perceived health.

Known-groups construct validity^9^ of the EQ- 5D self-classifier and EQ-VAS was examined by testing ten priori hypotheses based on the literature or clinical experience. Hypotheses relating EQ-5D dimensions to other variables were:

1. Subjects reporting problems for any EQ-5D dimension would have lower scores for all SF-36 scales;^10^
2. Subjects reporting problems for EQ-5D mobility, self-care, usual activities or pain/discomfort dimensions would have larger score reductions for SF-36 physical functioning (PF), role limitation due to physical problem (RP) and bodily pain (BP) scales than for role limitation due to emotional problem (RE) and mental health (MH) scales;
3. Similarly, subjects reporting problems for the EQ-5D anxiety/depression dimension would have larger score reductions for SF-36 RE and MH scales;
4. Subjects with mobility, self care, or usual activities problems should have their lowest score in role limitation due to a physical problem;
5. Elderly (age ≥60 years) or
6. Subjects with chronic diseases should report more problems than other subjects.
  Hypotheses for the EQ-VAS were:
7. EQ-VAS scores would be higher in subjects reporting better global health measured using a 5-point scale (i.e lower score on the first question of the SF-36);^11^,^12^
8. EQ-VAS scores would correlate negatively with increasing age;^12^,^13^
9. Females will report lower (worse) EQ-VAS scores;
10. Subjects with chronic disease will report lower (worse) EQ-VAS scores than those without.

Hypothesized trends were tested as appropriate depending on the type of data and distribution using the chi-square, the Fisher exact test, *t* test, the Mann-Whitney test, and Pearson or Spearman correlation coefficients. To minimize false-positive tests of significance, a significance level of *P*<.01^14^ should be used as a criterion for hypothesis fulfillment. Test-retest reliability of EQ-5D dimensions was investigated using the Cohen κ. According to Landis and Koch,^15^ κ coefficients of less than 0.0 are poor, 0.0 to 0.20 are slightly poor, 0.21 to 0.40 are fair, 0.41 to 0.60 are moderate, 0.61 to 0.80 are substantial, and 0.81 to 1.00 are almost perfect. Data were analyzed with SPSS for Windows (version 9, SPSS Inc, USA).

## RESULTS

During the study period, 200 subjects were asked to participate and only 14 refused. The main reason behind refusal was lack of time. The demographic and clinical characteristics of those who refused to participate were similar to the study subjects. One hundred eighty-six subjects completed the baseline questionnaires. Table 1 shows the general characteristics of the subjects. Table 2 shows distribution of responses to EQ-5D dimensions. There was only one missing item from the self care and the usual activities dimensions indicating the practicality and simplicity of the translated version. Among five dimensions of the EQ-5D, the proportion of having any problem was highest for anxiety/depression with 102 (55%) subjects reporting having moderate or extreme problems. The mean EQ-VAS score was 72.2 (SD 15.5).

**Table 1:** General characteristics of the study subjects.

	n (%)
Mean age (SD, range)	45.3 (15.9, 19-77)

Age ≥60 years	45 (24.2)

Female	87 (46.8)

Participants with a chronic disease	95 (51.1)
Education level	
Not educated	7 (3.8)
Primary	39 (21.0)
Secondary	23 (12.4)
University/college*	113 (60.8)
Missing data	4 (2.2)

Marital status	
Single	49 (26.3)
Married	129 (69.4)
Widowed	5 (2.7)
Missing data	3 (1.6)

Profession	
Employed	62 (33.3)
Unemployed	3 (1.6)
University student	49 (26.3)
Housewife	53 (28.5)
Retired	16 (8.6)
Missing data	3 (1.6)

* Includes those who are still university students, see under profession.

**Table 2:** Distribution of responses to EQ-5D dimensions.

EQ.5D dimension	Response (%)		
	No problem	Moderate problem	Extreme problem
Mobility	127 (68.3)	58 (31.2)	1 (0.5)
Self care	172 (92.5)	12 (6.3)	1 (0.5)
Usual activities	125 (67.2)	53 (28.5)	8 (4.3)
Pain/discomfort	87 (46.8)	90 (48.4)	9 (4.8)
Anxiety/depression	84 (45.2)	93 (50)	9 (4.8)

Cronbach's α was 0.75 indicating that the EQ-5D has an acceptable internal consistency.

Fifty-two subjects (28.0%) participated in the follow-up telephone interview, with a 3-week median interval (interquartile range: 2 to 4 weeks). Cohen's κ values for EQ-5D mobility, self care, usual activities, pain/discomfort and anxiety/depression items were 0.66, 1.0, 0.48, 0.66, and 0.48 respectively (*P*≤.001 for all dimensions). Intraclass correlation coefficient for the EQ-VAS between the two periods was 0.78.

All of the four hypotheses relating EQ-5D dimensions to SF-36 scales were fulfilled (Tables 3 and 4). Subjects reporting moderate or extreme problems for EQ-5D dimensions had lower SF-36 scores than those without such problems. Similarly subjects reporting problems for EQ-5D mobility, self-care, usual activities or pain/discomfort dimensions had larger score reductions for SF-36 PF, RP and BP scales than for RE and MH scales. When subjects were grouped by their responses to the EQ-5D anxiety/depression dimension, the difference in scores for the SF-36 MH and RE scales was larger than that for all other scales. In addition, subjects with mobility, self care, or usual activities problems had their lowest score in role limitation due to physical problem. Elderly participants (n=45) and those with at least one chronic medical problem (n=95) had significantly reported more problems in all of the EQ-5D dimensions apart from anxiety/depression (Table 4).

**Table 3:** Mean SF-36 scores for subjects in different EQ-5D dimensions (hypothesis 1 to 4).

EQ-5D dimension	N	PF mean (SD)	RP mean (SD)	BP mean (SD)	GH mean (SD)	VI mean (SD)	SF mean (SD)	RE mean (SD)	MH mean (SD)
Mobility									
No problem	127	89.1 (11.8)^a^	79.7 (33.6)^a^	80.4 (19.9)^a^	66.8 (14.9)^a^	59.4 (19.6)^a^	79.7 (20.5)^a^	68.7 (41.1)^b^	67.4 (19.6)
With problem	59	50.2 (24.5)	26.7 (35.6)	49.6 (20.8)	46.2 (15.2)	44.1 (19.6)	56.4 (24.0)	53.1 (44.7)	63.2 (18.6)

Self care									
No problem	172	80.5 (20.7)^a^	67.0 (40.8)^a^	73.2 (23.4)^a^	61.9 (16.6)^a^	56.1 (20.4)^a^	74.5 (22.3)^a^	65.3 (42.6)	66.4 (19.5)
With problem	13	26.9 (21.6)	11.5 (21.9)	37.8 (19.7)	36.9 (18.3)	33.8 (14.3)	41.8 (29.9)	64.2 (44.2)	62.5 (17.4)

Usual activity									
No problem	125	88.4 (13.3)^a^	82.0 (31.2)^a^	81.3 (19.0)^a^	67.2 (14.6)^a^	60.0 (19.7)^a^	80.6 (19.8)^a^	69.6 (40.6)^c^	67.2 (19.7)
With problem	60	53.9 (25.4)	23.3 (34.1)	49.0 (21.0)	46.7 (15.5)	43.8 (18.5)	56.2 (23.6)	52.3 (45.3)	63.5 (18.3)

Pain/discomfort									
No problem	87	90.5 (12.1)^a^	84.8 (28.6)^a^	86.7 (16.4)^a^	69.2 (13.6)^a^	63.2 (19.2)^a^	82.3 (18.1)^a^	68.2 (42.2)	68.1 (19.5)
With problem	99	64.8 (26.8)	43.7 (42.9)	45.6 (22.2)	52.4 (17.3)	47.0 (19.2)	63.6 (25.5)	60.0 (43.1)	64.4 (19.1)

Anxiety/depression									
No problem	84	81.1 (19.5)^b^	73.2 (39.9)^c^	78.0 (21.8)^a^	65.4 (14.8)^a^	63.2 (17.3)^a^	80.3 (21.7)^a^	81.8 (32.9)^a^	76.0 (14.2)^a^
With problem	102	73.3 (28.0)	56.4 (42.3)	64.6 (25.5)	56.1 (18.9)	47.5 (20.8)	60.9 (24.2)	47.0 (44.5)	52.0 (19.2)

PF: physical functioning, RP: role limitation due to physical problem, BP: bodily pain, GH: general health, VI: vitality, SF: social functioning, RE; role limitation due to emotional problem, MH: mental health;

a *P*<.001;

b *P*<.05;

c *P*<.01.

**Table 4:** Relationships between responses to EQ-5D dimension and other variables (hypothesis 5 and 6).

EQ-5D dimension vs other variables	N (%) with mobility problems	N (%) with self care problems	N (%) with usual activities problems	N (%) with pain/discomfort problems	N (%) with anxiety/depression problems
Age ≥60	26 (57.8)^a^	9 (20)^a^	24 (54.5)^a^	36 (80)^a^	22 (48.9)
Age <60	32 (22.9)	4 (2.9)	35 (25)	62 (44.3)	79 (56.4)

Participants with chronic problems	51 (53.7)^a^	12 (12.6)^b^	44 (46.8)^a^	71 (74.7)^a^	56 (58.9)
Participants without chronic problems	8 (8.8)	1 (1.1)	16 (17.6)	28 (30.8)	46 (50.5)

a *P*<.001;

b *P*<.01

On the other hand, all four hypotheses for the EQ-VAS were fulfilled (Table 5). The EQ-VAS was positively correlated with global health (lower score on SF-1 indicates better health) and negatively correlated with increasing age. Participants with at least one chronic problem had significantly lower results than those without. Females scored lower on the EQ-VAS, but this was significant only at the 0.05 level.

**Table 5:** Correlation between EQ-VAS score and other variables (hypothesis 6-10).

EQ-VAS dimension vs other variables	Correlation	Mean (SD)
EQ-VAS score vs SF-1	−0.83^a^	NA

EQ-VAS score vs age	−0.54^a^	NA

EQ-VAS score vs gender		
Female	NA	76.7 (16.3)^b^
Male	NA	81.4 (14.4)

EQ-VAS score vs presence of chronic problems		
Participants with chronic problems	NA	71.5 (15.8)^a^
Participants without chronic problems	NA	87.2 (10.1)

a *P*<.001;

b *P*<.05

## DISCUSSION

This is the first report on the reliability and validity of the EQ-5D in Arabic. All of the ten priori hypotheses were fulfilled, suggesting that the translation has properties similar to those of other validated EQ-5D versions. Internal consistency of the instrument was also found to be acceptable. We also found evidence to support test-retest reliability of the EQ-5D self-classifier, with Cohen's κ being moderate to perfect (0.48-1.0). The κ values in our study were in general better than those reported in previous studies of subjects after stroke using EQ-5D (Cohen's κ: 0.63-0.80, 3-week, n=234)16 and in those with rheumatic diseases (Cohen's κ: 0.29-0.61, 1-week, n=52).^3^ The significant and reasonably high intraclass correlation coefficient obtained in the EQ-VAS reliability study (0.78) demonstrates that the EQ-VAS is a feasible measure of self-reported health.

The results have demonstrated that the major problem reported by 55% of participants was anxiety/depression. Anxiety and depression are commonly associated with the etiology of many diseases including asthma, diabetes and hypertension. This reuslt is very important in the view of the large prevalence of diabetes and hypertension in Jordan, which exceeds 25%.^17^

We have utilized the Arabic version of the EQ-5D in several clinical settings including diabetes, rheumatoid arthritis and allergic rhinitis; the instrument was found to be of high clinical value and was strongly correlated with the clinical indicators. This indicates that the Arabic version of the EQ-5D is externally valid. It should be noted that the participants have completed the EQ-5D with the help of a research assistant. EQ-5D is a very simple instrument; therefore we think there will not be any major difference in the results if the EQ-5D was self administered.

One study limitation was that we used a convenience sample, which may limit the generalizability of the results. This limitation was obvious as the sample was characterized by a high level of education. However; this study is a pilot investigation and we hope in the near future to conduct a population-based investigation. It would have been more appropriate to validate the EQ-5D using a similar utility measure rather then using the SF-36. However, in our case this was not possible as currently the SF-36 is the only available well validated and well translated generic QOL instrument in the Arabic language.
